# Editorial Comment from Dr Sekino and Dr Hinata to A case of metastatic treatment emergent small cell/neuroendocrine prostate cancer with BRCA2 mutation diagnosed by liver biopsy

**DOI:** 10.1002/iju5.12507

**Published:** 2022-07-15

**Authors:** Yohei Sekino, Nobuyuki Hinata

**Affiliations:** ^1^ Department of Urology, Graduate School of Biomedical and Health Sciences Hiroshima University Hiroshima Japan

Treatment‐emergent small cell/neuroendocrine prostate cancer (t‐SNPC) mainly occurs in the advanced or metastatic castration‐resistant prostate cancer that is caused by the transformation of prostate adenocarcinoma after androgen deprivation therapy (ADT).^1^ t‐SNPC is an aggressive disease with a poor prognosis. The ADT treatment is ineffective, and the combined chemotherapy strategy of etoposide plus cisplatin/carboplatin was effective for patients with t‐SNPC.^2^ Therefore, early diagnosis and treatment play an important role in the survival in the patient with t‐SNPC. This case report by Naiki *et al*. showed that metastatic t‐SNPC with BRCA2 mutation diagnosed by liver biopsy.^3^

In this case report, the patient was diagnosed as t‐SNPC by the pathological findings of liver metastasis. The incidence of t‐SNPC may rise with the widespread use of new androgen receptor‐targeted therapies.^1^ However, the incidence of t‐SNPC may be underestimated because there is currently no criteria to perform a biopsy for t‐SNPC. Additionally, the secondary biopsies are not routinely performed, leading to miss the opportunity to diagnose t‐SNPC. The clinicians should consider the second biopsies when examining patients with the atypical and aggressive clinical courses.

In this case report, FoundationOne® CDx (Foundation Medicine, Cambridge, MA, USA) was used for cancer‐related gene profiling using liver tumor specimen, and a BRCA2 mutation was identified. BRCA2 is a key player in DNA damage repair (DDR). A recent study reported that DDR aberrations were rare in t‐SNPC. In contrast, alterations in DDR were identified in patients with de novo NEPC.^4^ In this case report, BRCA2 mutation was found after chemotherapy treatment. However, there was no time to use PARP inhibitor treatment because of the rapid and aggressive clinical course. This indicates that clinicians should consider gene profiling examinations, such as FoundationOne® CDx, at an early stage for the treatment options when seeing patients with t‐SNPC.

## Conflict of interest

The authors declare no conflict of interest.
